# Supplementary material for: The Glass-Transition Temperature of Supported PMMA Thin Films with Hydrogen Bond/Plasmonic Interface
Source: Polymers (Basel). 2019 Apr 2;11(4):601. doi: 10.3390/polym11040601 (PMC6523344; doi:10.3390/polym11040601)
Supplement: Supplementary file 1 [file polymers-11-00601-s001.pdf]

Supplementary Material

The Glass-Transition Temperature of Supported Polymer Thin Films with Hydrogen Bond/Plasmonic Interface

Jiayao Chen, Jing Li, Lirong Xu, Wei Hong, Yuzhao Yang,* and Xudong Chen*

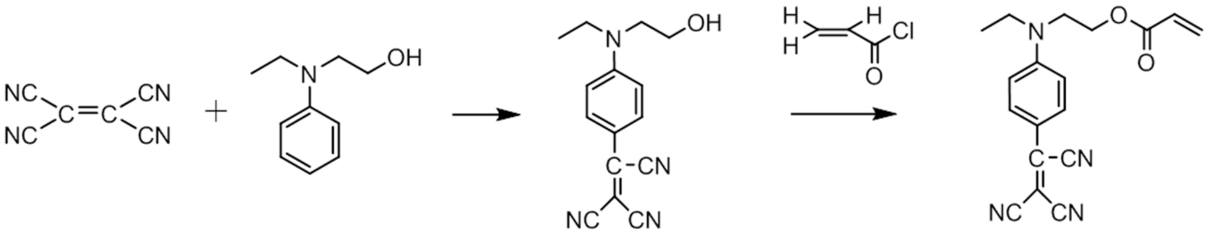

Figure S1. The synthesis route of the TC1-labeled methacrylate monomer.

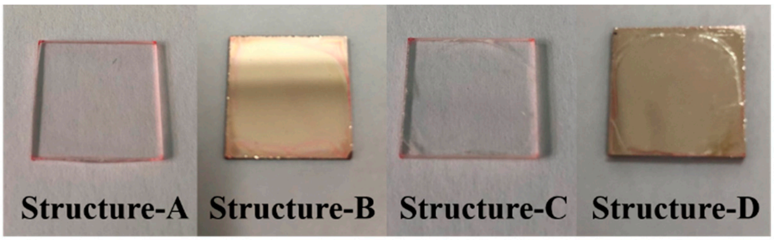

Figure S2. Photographs of four structures of TC1-labeled PMMA denoting as structure-A (quartz/PMMA-TC1), structure-B (quartz/PMMA-TC1/bulk layer), structure-C (quartz/Ag/PMMA-TC1), and structure-D (quartz/Ag/PMMA-TC1/bulk layer).

The synthesis of the probe TC1, TC1-labeled MMA monomer and TC1-labeled PMMA were confirmed by NMR and FT-IR as follows (see in **Figure S3**).

^{1}H NMR of TC1: ^{1}H NMR (300 MHz, $CDCl_3$, δ) 8.05 (d, J = 9.4 Hz, 2H), 6.80 (d, J = 9.5 Hz, 2H), 3.92 (d, J = 5.6 Hz, 2H), 3.74-3.42 (m, 4H), 1.30 (dd, J = 7.7, 6.5 Hz, 3H).

^{1}H NMR of TC1-labeled MMA monomer: ^{1}H NMR (300 MHz, $CDCl_3$, δ) 8.06 (d, J = 9.4 Hz, 2H), 6.83 (d, J = 9.4 Hz, 2H), 6.07 (s, 1H), 5.61 (s, 1H), 4.38 (t, J = 6.1 Hz, 2H), 3.79 (t, J = 6.1 Hz, 2H), 3.59 (q, J = 7.1 Hz, 2H), 1.93 (d, J = 1.1 Hz, 3H), 1.29 (dd, J = 12.5, 5.3 Hz, 4H).

The TC1 molecules were successfully grafted to the PMMA as evidenced by the ^{1}H NMR spectra (Figure S2c). In addition, compared with TC1-labeled MMA monomer, the 2218

cm^{-1} in TC1-labeled PMMA would be inferred to the characteristic peak of neat TC1 according to literature,[S1] which would further illustrate the presence of TC1 molecule in PMMA.

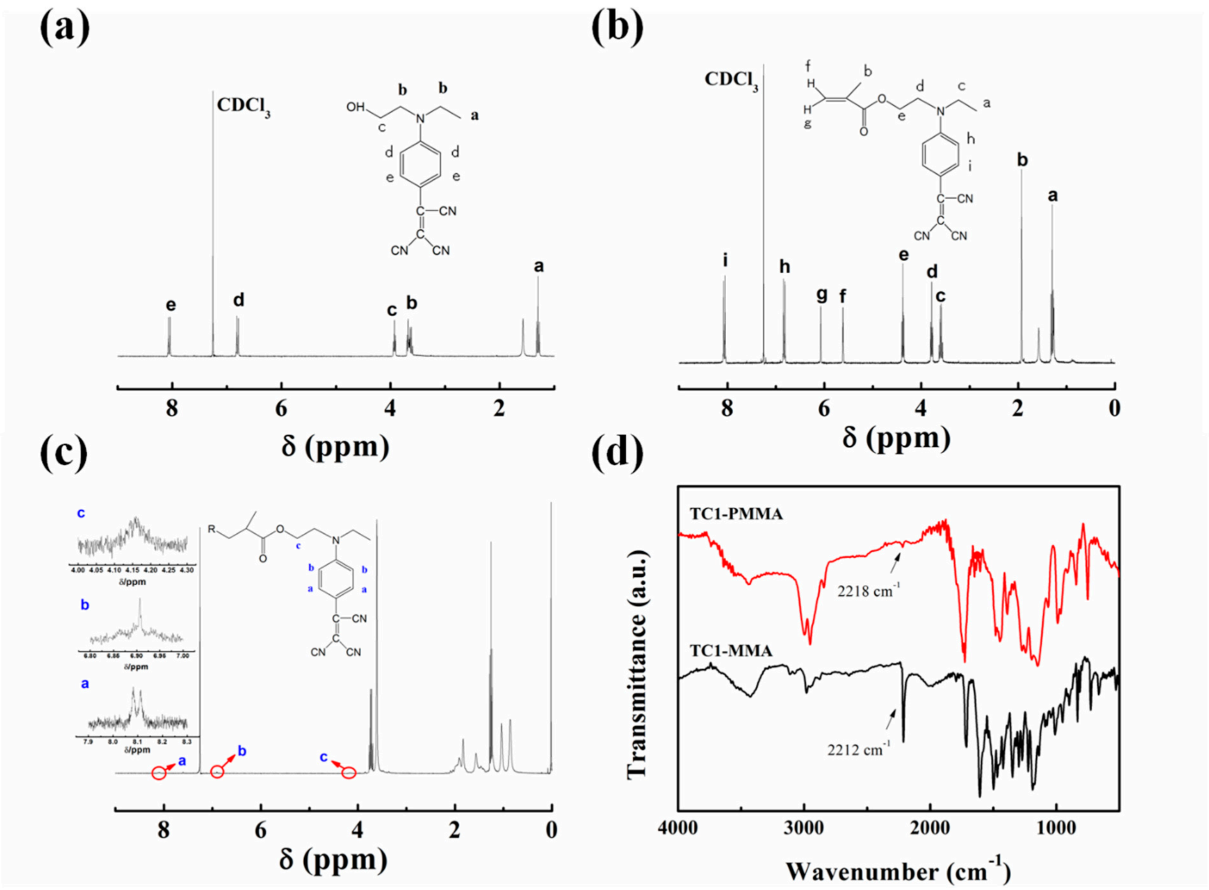

Figure S3. ^{1}H NMR (300 MHz, $CDCl_3$) spectra of (a) TC1, (b) TC1-labeled MMA monomer and (c) TC1-labeled PMMA (R for polymer repetitive unit). (d) The FT-IR spectra of TC1-labeled MMA monomer and TC1-labeled PMMA.

Table S1. The statistic information for fluorescence emission spectrum of TC1-labeled PMMA with thicknesses of 25, 110, 230 and 760 nm on quartz and silver substrate.

Batches	Peak positions of TC1-labeled PMMA on quartz substrate (nm)	Red-shifts of TC1-labeled PMMA on silver substrate with increasing film thickness (nm)
1	595	28 (606 to 578)
2	593	26 (609 to 583)
3	595	27 (598 to 571)
4	596	25 (598 to 573)
5	595	25 (598 to 573)
**Mean**	594.8	26.2
**Standard**	1.095	1.304

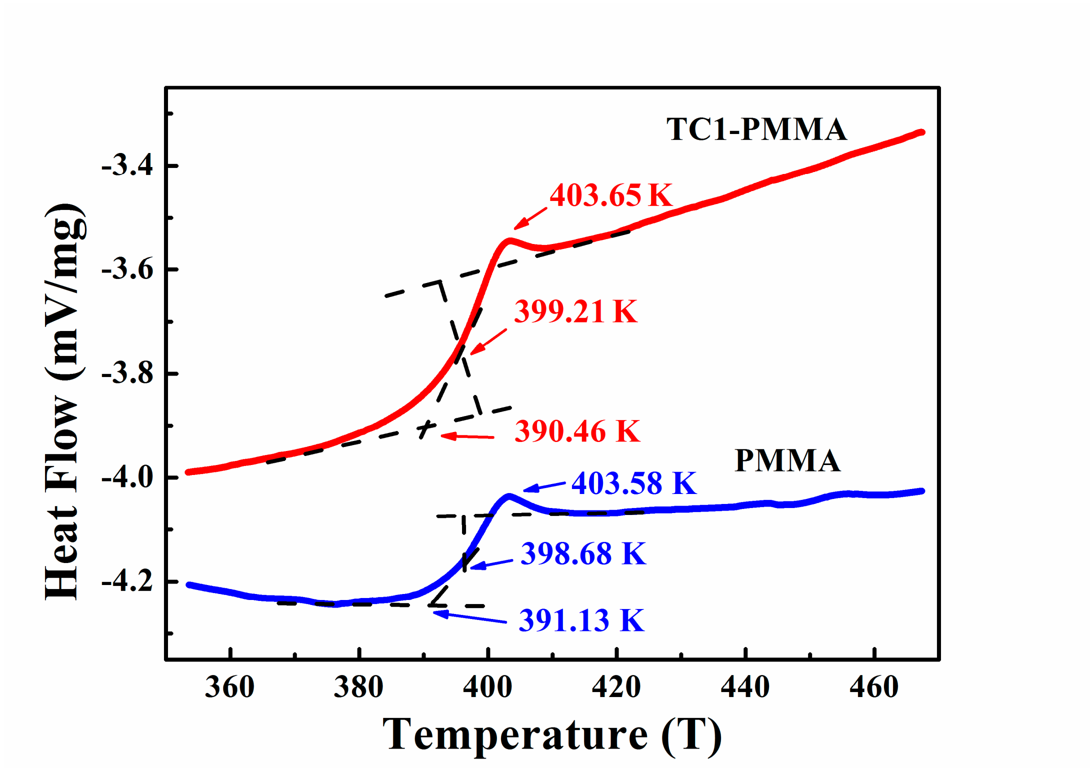

Figure S4. The DSC curve of PMMA and TC1-labeled PMMA bulk powder with T_gs.

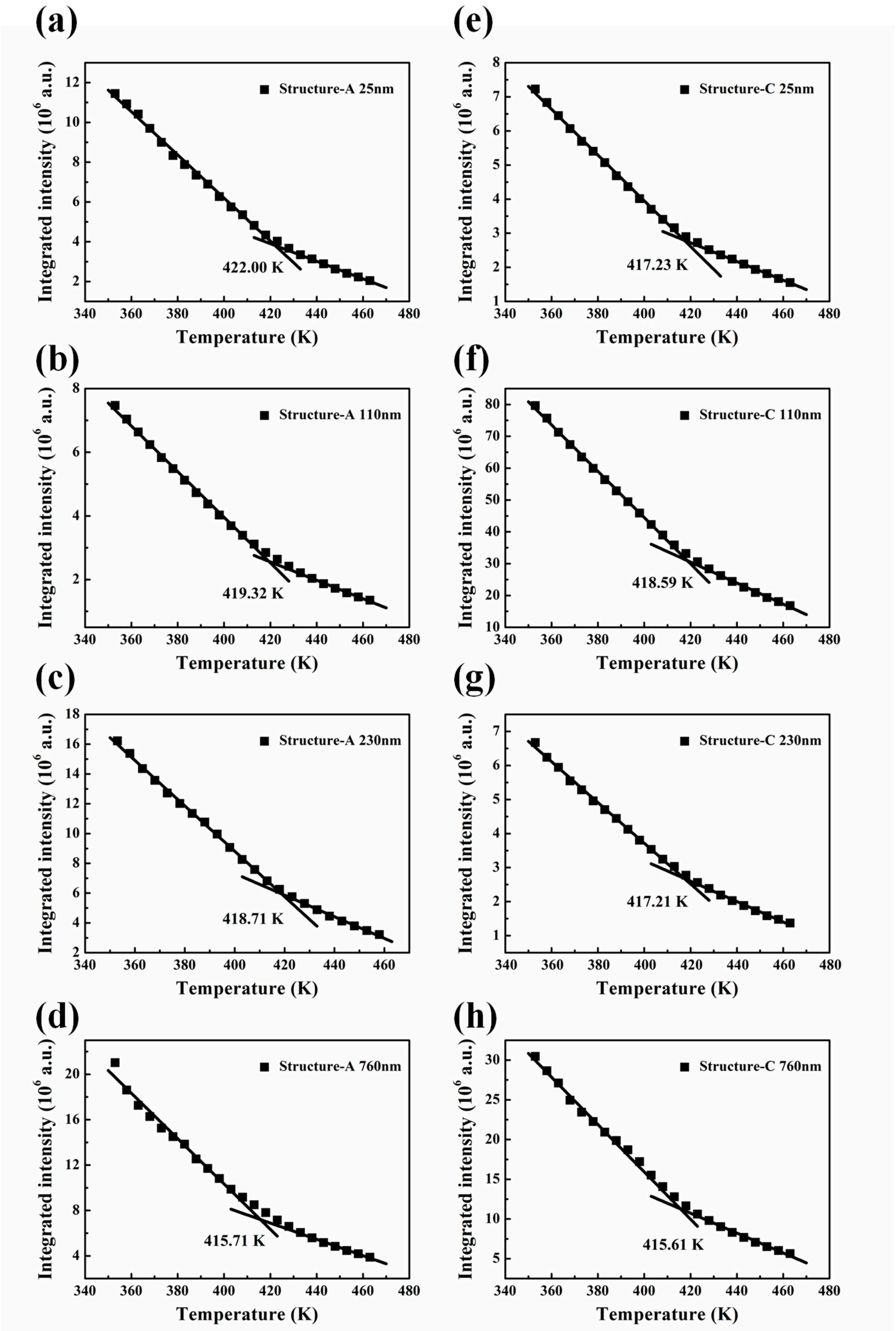

Figure S5. Temperature dependence of the integrated FL intensity of structure-A with thickness of (a) 25 nm, (b) 110 nm, (c) 230 nm and (d) 760 nm; structure-C with thickness of (e) 25 nm, (f) 110 nm, (g) 230 nm and (h) 760 nm.

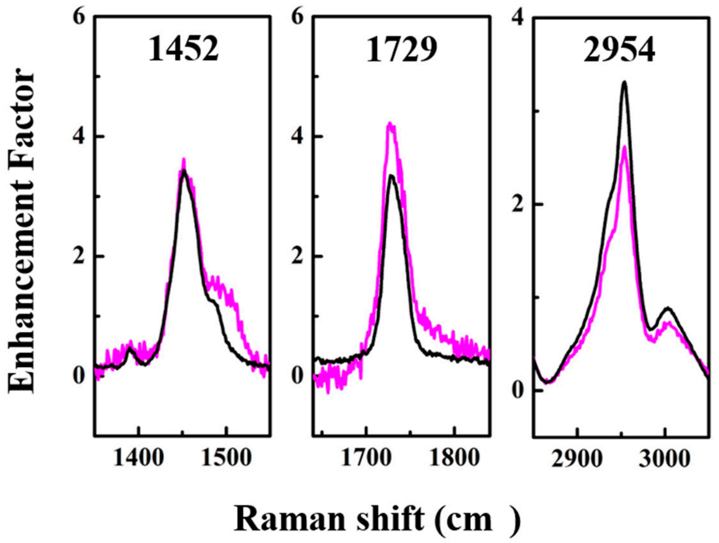

Figure S6. Raman enhancement factors of other batches of PMMA bulk film (760 nm, black line) and thin film (25 nm, red line) supported on silver substrates.

References

[S1] Ellison, C. J.; Mundra, M. K.; Torkelson, J. M. Impacts of polystyrene molecular weight and modification to the repeat unit structure on the glass transition-nanoconfinement effect and the cooperativity length scale. *Macromolecules* **2005**, *38*, 1767-1778.
